# Modified Gold Screen-Printed Electrodes for the Determination of Heavy Metals

**DOI:** 10.3390/s24154935

**Published:** 2024-07-30

**Authors:** Consuelo Celesti, Salvatore Vincenzo Giofrè, Claudia Espro, Laura Legnani, Giovanni Neri, Daniela Iannazzo

**Affiliations:** 1 Department of Engineering, University of Messina, Contrada Di Dio, 98166 Messina, Italy; espro@unime.it (C.E.); gneri@unime.it (G.N.); diannazzo@unime.it (D.I.); 2 Department of Chemical, Biological, Pharmaceutical and Environmental Sciences, University of Messina, Viale Ferdinando Stagno D’Alcontres 31, 98166 Messina, Italy; 3 Department of Biotechnology and Biosciences, University of Milano Bicocca, Piazza della Scienza 2, 20126 Milano, Italy; laura.legnani@unimib.it

**Keywords:** heavy metals, screen-printed electrodes, electrochemical detection, anodic stripping voltammetry

## Abstract

Screen-printed electrodes (SPEs) are reliable, portable, affordable, and versatile electrochemical platforms for the real-time analytical monitoring of emerging analytes in the environmental, clinical, and agricultural fields. The aim of this study was to evaluate the electrochemical behavior of gold screen-printed electrodes (SPGEs) modified with molecules containing amino (**Tr-N**) or α-aminophosphonate (**Tr-P**) groups for the selective and sensitive detection of the toxic metal ions Pb^2+^ and Hg^2+^ in aqueous samples. After optimizing the analytical parameters (conditioning potential and time, deposition potential and time, pH and concentration of the supporting electrolyte), anodic square wave stripping voltammetry (SWASV) was used to evaluate and compare the electrochemical performance of bare or modified electrodes for the detection of Hg^2+^ and Pb^2+^, either alone or in their mixtures in the concentration range between 1 nM and 10 nM. A significative improvement in the detection ability of Pb^2+^ ions was recorded for the amino-functionalized gold sensor **SPGE-N**, while the presence of a phosphonate moiety in **SPGE-P** led to greater sensitivity towards Hg^2+^ ions. The developed sensors allow the detection of Pb^2+^ and Hg^2+^ with a limit of detection (LOD) of 0.41 nM and 35 pM, respectively, below the legal limits for these heavy metal ions in drinking water or food, while the sensitivity was 5.84 µA nM^−1^cm^−2^ and 10 µA nM^−1^cm^−2^, respectively, for Pb^2+^ and Hg^2+^. The reported results are promising for the development of advanced devices for the in situ and cost-effective monitoring of heavy metals, even in trace amounts, in water resources.

## 1. Introduction

With the progress of industrial development, global environmental pollution has become more critical [1,2,3]. Heavy metal ions are among the most relevant water contaminants affecting terrestrial and aquatic ecosystems [4,5]. The foremost source of these heavy metal ions are cosmetics and their derivatives. Another source is chemicals from industrial or domestic waste [6]. Heavy metal ions are spread everywhere and are not able to be degraded. For this reason, these pollutants pose a threat to human health and the environment [7]. These ions are released from factories, accumulate in the biosphere, and then penetrate organisms through the food chain. Heavy metals such as mercury, cadmium, arsenic, and lead represent a risk to human health even at low concentrations since they react with the thiol group of proteins and thus enter cells, altering the biochemical life cycle [8]. Furthermore, high concentrations of silver and copper also have harmful effects [7,9,10]. These metals are extremely hazardous pollutants and are among the top ten on the ‘Priority List of Hazardous Substances of Toxic Substances and Disease Registry’ [11,12,13]. Disease Control and the European Union have listed heavy metals as priority substances and have instituted a list of permitted concentration limits [14]. The Joint Expert Committee on Food Additives of the Food and Agriculture Organization/World Health Organization and the International Agency for Research on Cancer evaluate the toxicity of heavy metals [15,16,17]. In recognition of the risks that heavy metal ions represent for the environment and for the human organism, it is particularly important to have an accurate and efficient detection technology. Exposure to lead and mercury specifically can cause health problems extending from stomach aches to brain damage. The U.S. Environmental Protection Agency (EPA) has set detection limits for these heavy metals, which are 15 µg/L and 0.6 µg/L for lead and mercury, respectively [18,19].

Several methods have been developed for the detection of heavy metal ions, such as atomic absorption spectroscopy [20,21], mass spectroscopy with inductively coupled plasma [22,23,24,25,26], neutron activation analysis [27,28], X-ray fluorescence spectrometry [29,30,31,32,33,34], and optical emission with inductively coupled plasma [23,35,36,37,38]. These technologies provide excellent detection limits and can measure several metal ions simultaneously. Nevertheless, spectroscopic techniques are expensive. Qualified staff can only use the instruments required for these techniques, involving complex pre-processing and sample analysis methods. Although optical techniques can exactly detect heavy metal ions, expensive heavy metals, pricey equipment, and complex operations are required. Additionally, these techniques are not well adapted for on-site detection [39,40]. Hence, efficient, low-cost, simple, and reliable detection technology is a crucial path to detect metal ions in situ [41,42,43,44,45].

Electrochemical detection can be applied to overcome the limitations of other methods. Electrochemical methods are easy to use, cheap, and reliable. In addition, these techniques can be implementable in the field, providing portability and rapid response to the on-site analysis of polluted samples. Modifying electrochemical sensors with specific substances can significantly improve their performance. For example, the loading with metal nanoparticles can accelerate the electron transfer rate between the analyte and the electrode. Semiconductor nanomaterials can enhance the efficiency of photochemical reactions and improve the detection performance of heavy metal ions [46,47,48]. The sensitivity and detection limit of electrochemical techniques can be improved by using several electrochemical sensors combined with different electrochemical techniques, which offer lower complexity, higher analysis speed, lower cost, and miniaturization capability with detection limits of ppb (part per billion) [49,50]. Therefore, these techniques have long been suggested as alternative strategies for the in situ monitoring of heavy metals.

Among the wide range of electrochemical techniques, anodic stripping voltammetry (ASV) has demonstrated the ability to sensitively detect most heavy metals, even in trace amounts [51]. Considering the relevance of miniaturization to improve field performance, screen-printed electrodes (SPEs) represent the key to developing new portable sensing systems in recent years. SPEs are simple, economical, very suitable for on-site analysis, and can be used to develop versatile (bio)sensors for a variety of applications, including clinical biomarkers, environmental monitoring, and agrifood sectors [51,52,53,54,55].

SPEs with gold working electrodes (SPGEs) are particularly suitable for a wide range of biosensing applications [56]. The easy gold surface functionalization with homobifunctional thiol-cleavable crosslinking reagents, such as the dithiobis (succinimidyl propionate linker (DSP), allows the development of self-assembled monolayers (SAMs) on the electrode surface, obtaining good reproducibility and high electron transfer rates [57].

In the present research, we wanted to investigate, for the first time, the thiol chemistry functionalization of SPGE for selective heavy metal detection. The working electrodes of SPGEs have been modified with the molecules **Tr-N** and **Tr-P** containing amino or α-aminophosphonate groups, which are able to recognize Pb^2+^ and Hg^2+^, respectively, in water. The selected ligands have previously demonstrated, through experimental and computational studies, the ability to selectively bind Hg^2+^ or Pb^2+^ ions, respectively, in mixtures of these and other competing ions [58]. These sensitive elements have been covalently linked to the electrodes using the cross-linker dithiobis(succinimidylpropionate) (DSP), which is able to form stable Au-S bonds [59], and covalently link the sensitive elements through the two NHS-activated esters to obtain **SPGE-N** and **SPGE-P**. After the optimization of the chemical and instrumental conditions, type of supporting electrolyte, deposition potential, pH, and time, square wave stripping anodic voltammetry (SWASV) was chosen as the electrochemical method for the detection of Hg^2+^ and Pb^2+^ alone or in their mixtures. **SPGE-N** and **SPGE-P** demonstrated a good selectivity towards Pb^2+^ and Hg^2+^ ions, respectively, with a limit of detection of 0.41 nM for Pb^2+^ and 35 pM for Hg^2^, which are below the legal limits in drinking water or food. The obtained results, compared with those obtained from bare gold electrodes, highlight the improved selectivity and sensitivity of the modified sensors towards the selected toxic ions. This study can pave the way for an innovative, reliable, and, at the same time, accessible route for monitoring water quality using simply modified SPEs and anodic stripping voltammetry.

## 2. Materials and Methods

### 2.1. Chemicals and Materials

All reagents and solvents were acquired from Sigma Aldrich (St. Louis, MO, USA) and used without any further purification. Lead nitrate (purity ≥ 99.95%) and mercury nitrate monohydrate (purity ≥ 99.99%) were used to prepare aqueous solutions of the heavy metals concerned. The acetate buffer was prepared using a 0.1 M solution of acetic acid and NaOH to obtain the desired pH. Double-distilled water was used for the entire experimental part. All chemicals and reagents were of analytical grade and were used without additional purification, unless otherwise stated. SPGEs were purchased from Metrohm-DropSens (www.dropsens.com). NMR spectra were recorded at 500 MHz; chemical shifts are given in parts per million, using trimethylsilane (TMS) as the internal standard. Thin-layer chromatography was performed on aluminum plates pre-coated with Merck 60-F254 silica gel. Preparative separations were carried out by flash column chromatography using 0.063–0.200 mm Merck silica gel. Infrared spectra were obtained with a Spectrum Two FT-IR spectrometer (PerkinElmer Inc., Waltham, MA, USA) using the ATR method in the range 4000–500 cm^−1^. The percentage of elemental composition (including carbon, nitrogen, hydrogen, sulfur, and oxygen) in the samples was also determined with the CHNS-O EMA 502 VELP (NY, USA).

### 2.2. Synthesis of the Sensitive Elements ***Tr-N*** and ***Tr-P***

#### 2.2.1. Synthesis of **Tr-N**

**Scheme 1:**
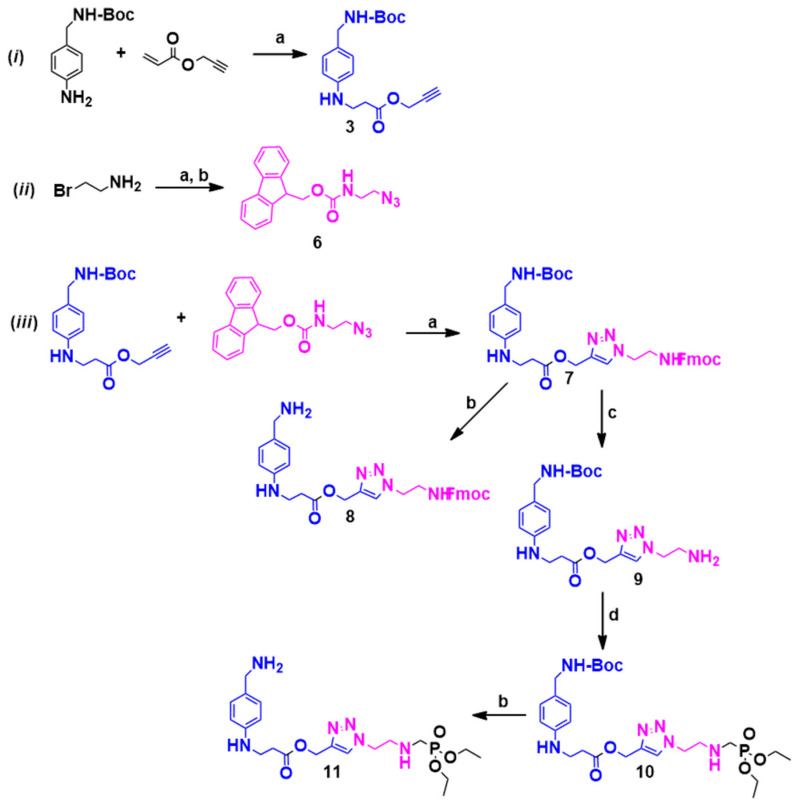
Reagents and conditions: (**i**) (a) HFIP, 48 h at r.t., yield 90%; (**ii**) (a) NaN_3_, H_2_O, 80 °C, 24 h, yield 88%; (b) Fmoc-Cl, KOH, Acetone, 12 h, t.a. yield 85%; (**iii**) (a) CuSO_4_·5H_2_O, sodium ascorbate, TEA, 12 h, t.a., yield 85%; (b) trifluoroacetic acid, DCM, 1h, t.a., yield 80%, (c) piperidine, CH_2_Cl_2_, overnight, yield 90%; (d) CH_2_O (37 wt%), triethyl phospite, 16 h, reflux, yield 60%.

As depicted in Scheme 1, the synthesis of **Tr-N** involved the click chemistry reaction of prop-2-yn-1-yl-3-((4-(((tert-butoxycarbonyl)amino)methyl)phenyl)amino) propanoate **3**, obtained from the Michael-type reaction of **1** and **2**, with (9H-fluoren-9-yl)methyl (2-azidoethyl)carbamate **5**, derived from 2-bromoethylamine **4**, and the subsequent Boc-deprotection, following the procedure reported below.

Synthesis of prop-2-yn-1-yl 3-((4-(((tert-butoxycarbonyl)amino)methyl)phenyl)amino) propanoate (**3**). To a solution of tert-butyl 4-aminobenzylcarbamate **1** (1 g, 4.50 mmol) in HFIP (10 mL), we added propargyl acrylate **2** (1 mL, 9 mmol) at r.t. The reaction was allowed to react at room temperature for 48 h. After this time, the solvent was removed under reduced pressure and the residue was purified by flash chromatography on silica gel (CHCl_3_/MeOH 95:5) to obtain the pure product **3** as a yellow solid (see Supplementary Materials for NMR data and elemental analyses).

Synthesis of (9H-fluoren-9-yl)methyl (2-azidoethyl)carbamate (**5**). 2-Bromoethylamine **4** (6.161 g, 30 mmol) was added to a solution of sodium azide (6.20, 95 mmol) in 20 mL of distilled water. The reaction was conducted under reflux at 80 °C for 24 h. The reaction mixture was cooled to 0 °C, and KOH (7.99 g, 142 mmol) was added. The reaction was allowed to react at 0 °C and was extracted with ethyl acetate (4 × 35). To a suspension of fluorenylmethoxycarbonyl chloride (1531 g, 5.92 mmol) in acetone (20 mL), a solution of 2-azido-ethylamine (509 mg, 5.92 mmol) and potassium carbonate (818 mg, 5.92 mmol) was added in 15 mL of distilled water. The reaction was allowed to react overnight at room temperature. After this time, the solvent was removed under reduced pressure, and the residue was first extracted with ethyl acetate (4 × 50) and then purified by flash chromatography on silica gel (CHCl_3_/MeOH 95:5) to obtain **5** as a white solid (see Supplementary Materials for NMR data and elemental analyses).

Synthesis of (1-(2-((((9H-fluoren-9-yl)methoxy)carbonyl)amino)ethyl)-1H-1,2,3-triazol-4-yl)methyl 3-((4-(((tert-butoxycarbonyl)amino)methyl)phenyl)amino)propanoate (**6**). To a dispersion of ethynyl 2-((4-((tert-butoxycarbonyl)methyl)phenyl)amino)acetate 3 (181 mg, 0.54 mmol), (9H-fluoren-9-yl)methyl (2-azidoethyl)carbamate **5** (168 mg, 0.54 mmol), and triethylamine (0.08 mL, 0.54 mmol) in acetonitrile (20 mL) and distilled water (20 mL), copper sulfate (34 mg, 0.135 mmol) and sodium ascorbate (53.49 mg, 0.27 mmol) were added. The reaction is left under stirring overnight at room temperature. After this time, the solvent was removed under reduced pressure, and the residue was purified by flash chromatography on silica gel (CHCl_3_/MeOH 95:5) to obtain the pure product **6** as a yellow oil (see Supplementary Materials for NMR data and elemental analyses).

Synthesis of (1-(2-((((9H-fluoren-9-yl)methoxy)carbonyl)amino)ethyl)-1H-1,2,3-triazol-4-yl)methyl 3-((4-(aminomethyl)phenyl)amino)propanoate (**Tr-N**). To a solution of (1-(2-((((9H-fluoren-9-yl)methoxy)carbonyl)amino)ethyl)-1H-1,2,3-triazol-4-yl)methyl 3-((4-(((tert-butoxycarbonyl)amino)methyl)-phenyl)amino)-propanoate **6** (450 mg, 0.7 mmol) in CH_2_Cl_2_ (30 mL), trifluoroacetic acid (1.07 mL, 14 mmol) was added, and the mixture was allowed to react at room temperature overnight. After this time, the mixture was extracted with CH_2_Cl_2_ (4 × 40) and was purified by flash chromatography on silica gel (CHCl_3_/MeOH 95:5) to obtain the pure product **Tr-N** as a yellow oil (see Supplementary Materials for NMR data and elemental analyses).

#### 2.2.2. Synthesis of **Tr-P**

The synthesis of **Tr-P** was performed, starting from compound **6** by Fmoc-deprotection, a subsequent Mannich-type reaction of the free amino group with formaldehyde and triethyl phosphite, and finally by the cleavage of the Boc group. Below, the detailed procedure is reported.

Synthesis of (1-(2-aminoethyl)-1H-1,2,3-triazol-4-yl)methyl 3-((4-(((tert-butoxycarbonyl)amino) methyl)phenyl)amino)propanoate (**7**). To a solution of (1-(2-((((9H-fluoren-9-yl)methoxy)carbonyl)amino)ethyl)-1H-1,2,3-triazol-4-yl)methyl 3-((4-(((tert-butoxycarbonyl)amino)methyl)-phenyl)amino)-propanoate **6** (778 mg, 1.214 mmol) in CH_2_Cl_2_ (30 mL), piperidine (0.07 mL, 1.214 mmol) was added, and the mixture was allowed to react at room temperature overnight. After this time, the solvent was removed under reduced pressure and the residue was purified by flash chromatography on silica gel (CHCl_3_/MeOH 9:1) to obtain the pure product **7** as a yellow oil (see Supplementary Materials for NMR data and elemental analyses).

Synthesis of (1-(2-(((diethoxyphosphoryl)methyl)amino)ethyl)-1H-1,2,3-triazol-4-yl)methyl 3-((4-(((tert-butoxycarbonyl)amino)methyl)phenyl)amino)propanoate (**8**). To a solution of (1-(2-aminoethyl)-1H-1,2,3-triazol-4-yl)methyl 3-((4-(((tert-butoxycarbonyl)amino)methyl)phenyl)amino)propanoate **7** (210 mg, 0.5 mmol) in ethanol (3 mL), triethyl phosphite (0.125 mmol, 0.022 mL) and formaldehyde solution 37% (0.25 mmol, 0.02 mL) was added, and the mixture was allowed to react at reflux for 16 h. After this time, the solvent was removed under reduced pressure and the residue was purified by flash chromatography on silica gel (CHCl_3_/MeOH 95:5) to obtain the pure product **8** as a yellow oil (see Supplementary Materials for NMR data and elemental analyses).

Synthesis of (1-(2-(((diethoxyphosphoryl)methyl)amino)ethyl)-1H-1,2,3-triazol-4-yl)methyl 3-((4-(aminomethyl)phenyl)amino)propanoate (**Tr-P**). To a solution of (1-(2-(((diethoxyphosphoryl)methyl)amino)ethyl)-1H-1,2,3-triazol-4-yl)methyl 3-((4-(((tert-butoxycarbonyl)amino)methyl)phenyl)amino)propanoate **8** (100 mg, 0.176 mmol) in CH_2_Cl_2_ (10 mL), trifluoroacetic acid (0.29 mL, 3.517 mmol) was added, and the mixture was allowed to react at room temperature overnight. After this time, the volatiles were removed in vacuo. The crude was diluted with CH_2_Cl_2_ and sat. aq. NaHCO_3_. The mixture was extracted with CH_2_Cl_2_ (4 × 30) and was purified by flash chromatography on silica gel (CHCl_3_/MeOH 9:1) to obtain the pure product **Tr-P** as a yellow oil (see Supplementary Materials for NMR data and elemental analyses).

### 2.3. Development of the Modified SPGEs (***SPGE-N*** and ***SPGE-P***)

To develop the **SPGE-N** and **SPGE-P** electrodes, the bare SPGEs were first functionalized using the reagent DSP as follows. A quantity of 10 mg of DSP was ultrasonically dissolved in 1 mL of DMSO. Then, 15 µL of the solution was dropped directly onto the surface of the gold working electrode and allowed to dry at room temperature until further use. Next, 10 mg of **Tr-N** or **Tr-P** was dissolved in 1 mL of DMSO. Then, 20 µL of the two solutions were deposited directly onto the surface of the DSP-SPGE gold working electrodes to obtain **SPGE-N** and **SPGE-P**, which were left to dry at room temperature until further use.

### 2.4. Electrochemical Tests

Electrochemical investigations were performed in a 5 mL beaker using the DropSens µStat 400 potentiostat and Dropview version 8400 software for data acquisition. Important instrumental parameters for SWV, such as the conditioning potential/time (Econd/tcond) and deposition potential/time (Edep/tdep), were each optimized and found to be 0.6 V/60 s and 1.1 V/240 s, respectively. After the pre-concentration step, analyses were carried out ranging from −1.1 to 0.8 V to achieve the analytical signal for the heavy metal concentration and subsequently to construct the calibration curves for each analyte. Initially, the response of the modified electrode without the presence of the ions was recorded. A stirring speed of 300 rpm was applied for the deposition of the ions prior to measurement. SWV was performed by adding microvolumes of standard aqueous solutions of metal ions to the electrochemical cell containing 5 mL of acetate buffer solution. To stabilize the SPEs, triplicate scans were performed for each new unit in the buffer solution before reliable measurements were made. To ensure the removal of metal ions from the working surface of the SPEs, a conditioning potential was applied at −1 V for 60 s before each measurement.

## 3. Results and Discussion

### 3.1. Synthesis of ***Tr-N*** and ***Tr-P***

The synthetic strategy for the development of the sensitive elements **Tr-N** and **Tr-P** to be loaded on the working electrode of SPGEs involves, as a key step, the formation of the 1,4-disubsituted-1,2,3-triazole **6** by Huisgen 1,3-dipolar cycloaddition reaction of the propargyl derivative 3 and azide 5, as depicted in Scheme 1 [60,61]. Compound 3 was obtained by a Michael-type addition reaction between the free amino group of tert-butyl 4-aminobenzylcarbamate 1 and the terminal double bond present in propargyl acrylate 2 (path *i*), while the reaction of 2-bromoethylamine 4 with sodium azide followed by the protection of the free amino group with fluorenylmethoxycarbonyl chloride (Fmoc) provided azide 5 (path *ii*). The Cu(II)-catalyzed 1,3-dipolar cycloaddition of 3 and 5, performed in the presence of ascorbate and in mild reaction conditions, afforded the triazole derivative **6** in a nearly quantitative yield (path *iii*). The sample **Tr-N** was then obtained in a quantitative yield by the deprotection of the Boc group using TFA at room temperature. For the synthesis of the **Tr-P** sample, the cleavage of Fmoc in compound **6** allowed the Mannich-type reaction of the free amino functionality of compound **7** with formaldehyde and triethyl phosphite to obtain compound **8**. Finally, the cleavage of the Boc group performed again using TFA afforded the **Tr-P** sample. All the synthesized compounds and intermediates have been purified and characterized, as reported in the experimental section.

### 3.2. Synthesis of the Ligand-Modified SPGE

**Figure 1:**
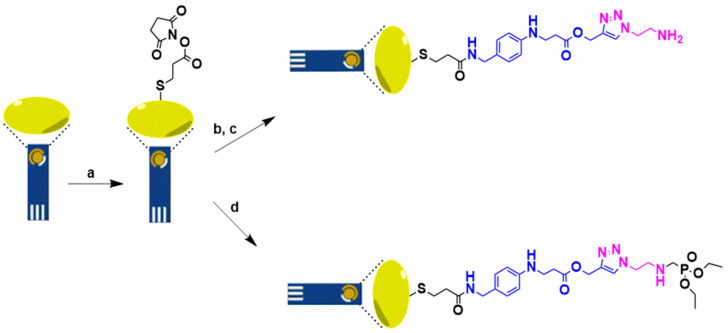
Application of the chelating linker on the SPGE. Reagents and conditions: (a) DMSO, 48 h; (b) Piperidine/H_2_O, 1 h; (c) **Tr-N**, 48 h; (d) **Tr-P**, 48 h.

The bare SPGEs were easily functionalized using the Lomant’s reagent as follows to obtain **SPGE-N** and **SPGE-P** electrodes, respectively (Figure 1). A quantity of 10 mg DSP (Lomant’s reagent) was ultrasonically dissolved in 1 mL of DMSO. Then, 15 µL of the solution was dropped directly onto the surface of the gold working electrode and allowed to dry at room temperature until further use. Next, 10 mg of samples 8 and 9 were dissolved in 1 mL of DMSO, respectively. Then, 20 µL of the two solutions were deposited directly onto the surfaces of the gold working electrodes (SPGEs) previously modified with DSP and left to dry at room temperature until further use.

**Figure 2:**
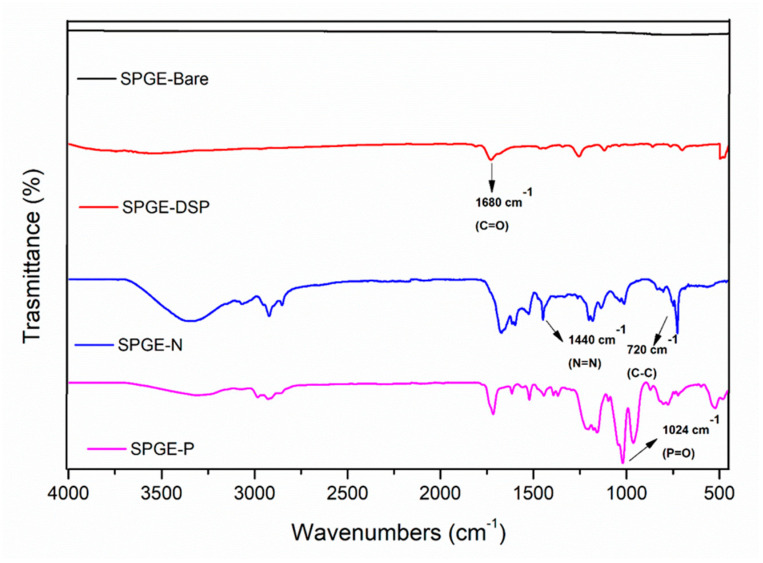
ATR-FTIR spectra of SPGE, SPGE-DSP, **SPGE-N**, and **SPGE-P**.

The Fourier transform infrared spectroscopy was used to verify the effectiveness of the chemical modification on the work electrode of SPGE. As shown in Figure 2, the FTIR spectra of SPGE showed no peaks. After modification with DSP, the absorbance peaks shown in the SPGE-DSP spectrum at 1786 cm^−1^ and 1740 cm^−1^ indicate the representative symmetric and asymmetric carbonyl stretches (respectively) of the NHS ester. Furthermore, the peak at 1212 cm^−1^ confirms the presence of the asymmetrical C-N-C stretch of the NHS ester, while the peak at 1069 cm^−1^ can be identified as the N-C-O stretch of succinimide. Finally, the peak at 1170 cm^−1^ indicates the carbonyl stretch of the ester. The presence of these peaks confirms the chemisorption of DSP on the gold surface. The electrode modification with DSP significantly changed the IR spectrum of the gold substrate; this sample shows the diagnostic peak at 1680 cm^−1^ due to the carbonyl stretch of amide moiety. The spectrum of **SPGE-N** shows the representative absorbance of the triazole ring at 1440 cm^−1^ due to the N=N stretching, while, for **SPGE-P**, the diagnostic peak at 1024 cm^−1^ due to the P=O stretching can be observed.

### 3.3. Electrochemical Properties of Bare SPGE, ***SPGE-N***, and ***SPGE-P***

**Figure 3:**
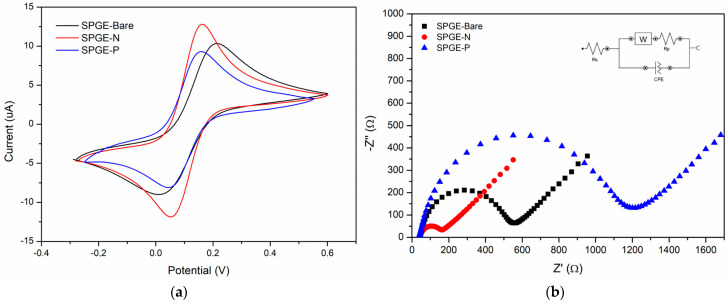
(**a**) Cyclic voltammogram of SPGE-Bare, **SPGE-N**, and **SPGE-P** in 0.01 M PBS (pH 7.4), scan rate 50 mV/s. (**b**) EIS of SPGE-Bare, **SPGE-N**, and **SPGE-P** in a solution containing 5 mM [Fe(CN)_6_]^3−/4−^ and 0.1 M KCl with a frequency range from 0.1 Hz to 0.1 MHz.

The electrochemical characteristics of modified SPGEs were evaluated by CV and EIS and were compared with those obtained from SPGEs (Figure 3). For cyclic voltammetry (Figure 3a), the voltage applied to the working electrode was run from −0.3 V to +0.6 V at a scan rate of 50 mV/s in a 5 mM solution of K_3_[Fe(CN)_6_] in PBS. A pair of redox peaks was recorded, underlining the electron transfer rate at the electrode–electrolyte interface.

These results were also demonstrated by EIS analysis; as shown in Figure 3b, SPGE shows a larger semicircle (Rct = 488 Ω) compared to **SPGE-N** (Rct = 124 Ω). These values indicate a decrease in the resistance of the modified electrode, greatly improving the ability of the modified sensor (**SPGE-N**) to perform the oxidation and reduction of the analytes. As for **SPGE-P**, this shows a larger semicircle than SPGE (Rct = 1110 Ω). The observed differences in RCT values can be rationalized considering the different chemical functionalization of the modified SPGEs. Since the main differences between **SPGE-P** and **SPGE-N** is the presence of amino groups (in **SPGE-N**) or sterically bulky phosphonic groups (in **SPGE-P**), it is conceivable that the active surface area, where the transfer of electrons takes place, is reduced for the modified electrodes, and, in particular, for **SPGE-P**, thus leading to higher RCT values for the modified sensors. Similar responses have also been observed in the literature for SPGE functionalized with consistent organic layers [62].

The determination of electrochemical active area (ECSA) values is essential to obtain a comparison of the performance between different electrodes and can provide useful indications of changes at the electrode interface after modification. Indeed, the observed improvement in the redox peak current values of ligand-based electrodes could be attributed to faster electron transfer kinetics and its higher electroactive surface area. The Randles–Sevcik formula was employed to find out the active surface area of SPGE, **SPGE-N**, and **SPGE-P** using K_4_[Fe(CN)_6_]/K_3_[Fe(CN)_6_] as the redox probe:

(1) $$Ip=2.69\times 10^{5}n^{3/2}AD^{1/2}Cv^{1/2}$$

where *Ip* is the peak current measured at the redox potential, *n* = 1 is the number of electrons transferred in the redox event, *A* = 0.126 cm^2^ is the surface area of the electrode (cm^2^), *D* = 7.6 × 10^−6^ cm^2^ s^−1^ is the diffusion coefficient (cm^2^ s^−1^), *C* is the concentration of the redox probe (ranging from 1 to 10 mM (10^−6^ to 10^−5^ mol cm^3^), and *v* = 0.05 is the potential scan rate (Vs^−1^). From the slope of Equation (1), the calculated areas for SPGE-Bare, **SPGE-N**, and **SPGE-P** were found to be 0.0063, 0.0076, and 0.0056 cm^2^, respectively, confirming that the electrode surface had been modified. 

**Figure 4:**
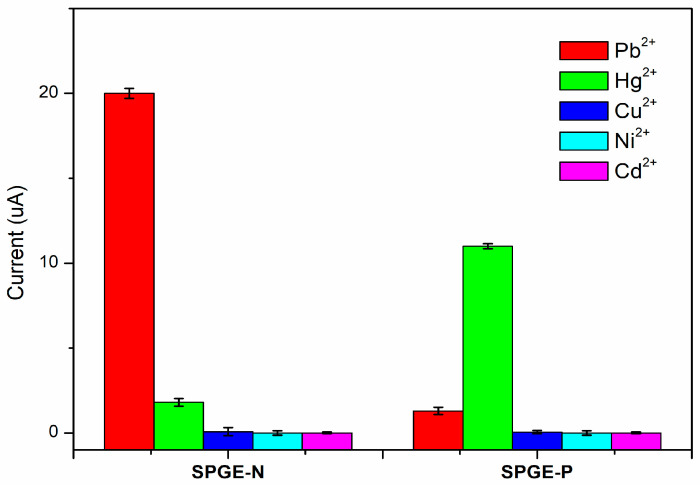
Electrochemical response to different heavy metal ions of **SPGE-N** and **SPGE-P**.

The selectivity of the electrodes was assessed by square wave voltammetry, testing them with different analytes, such as Ni^2+^, Cu^2+^, Cd^2+^, Pb^2+^, and Hg^2+^. As shown in Figure 4, a significant response for Pb^2+^ is recorded for the **SPGE-N** electrode at the nanomolar concentration, while no significant responses were recorded for Ni^2+^, Cu^2+^, Cd^2+^, and Hg^2+^ ions. Similarly, a significant response for Hg^2+^ were recorded for the **SPGE-P** electrode, while no relevant response was recorded for Ni^2+^, Cu^2+^, Cd^2+^, and Pb^2+^ ions. These results confirm the specific binding of the synthesized ligands with the target metal ions, guaranteeing the excellent selectivity of the functionalization procedures. 

In our previous study, we experimentally evaluated the chelating capacity of this type of system by means of ICP-MS analysis [58,63]. These studies revealed the interesting chelating behavior of the phosphonate system towards the Hg^2+^ ion and of the amine system for Pb^2+^ ions. Therefore, we detected Hg^2+^ and Pb^2+^ ions using SPGE and **SPGE-N** electrodes, selective towards Pb^2+^ ions, and **SPGE-P**, selective towards Hg^2+^ ions, immersed in a 0.1 M acetate buffer solution at pH 4.5 in the concentration range 1–10 nmol, following the SWASV method discussed in Section 2.4.

**Figure 5:**
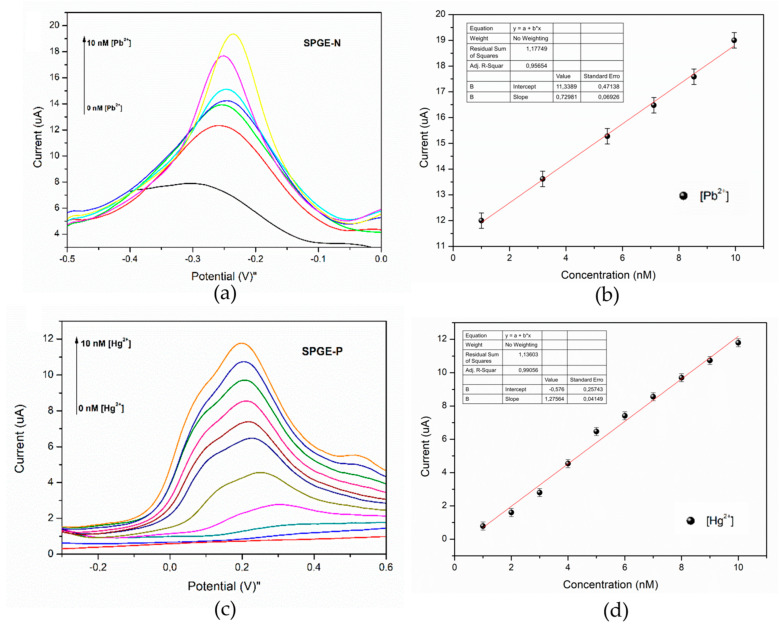
(**a**) SWASV of **SPGE-N** upon addition of Pb^2+^ ions (stripping peak at around −0.25 V) and (**b**) calibration graph at ion concentrations of 1–10 nmol. (**c**) SWASV of **SPGE-P** upon addition of Hg^2+^ ions (stripping peak at around +0.2 V) and (**d**) calibration graph at ion concentrations of 1–10 nmol.

Figure 5a shows the square-wave stripping anodic voltammetry (SWASV) analyses with the **SPGE-N** electrode, which were carried out ranging from −0.5 V to 0.0 V by adding small volumes of aqueous Pb^2+^ solutions (in the concentration range of 1–10 nM) into a cell containing a pH 4.5 buffer solution. Preliminary results obtained for **SPGE-N** show a detection capability towards Pb^2+^ ions more than 20 times higher than SPG. Upon the addition of the Pb^2+^ solution, the ion stripping peak at −0.20 V can be observed, while Figure 5b shows the corresponding calibration graph recorded in the concentration range of 1–10 nmol. Likewise, Figure 5c shows the SWASV analysis performed on the **SPGE-P** electrode, ranging from −0.3 V to 0.6 V, by adding aqueous solutions of Hg^2+^ ions (concentration range of 1–10 nM) in a pH 4.5 buffer solution, which revealed a higher detection capability of Hg^2+^ ions of more than 100 times that of bare SPGE (Figure 5c). Upon the addition of the Hg^2+^ solution, the ion stripping peak at 0.20 V can be observed, while Figure 5d shows the corresponding calibration graph performed in the concentration range of 1–10 nmol.

**Figure 6:**
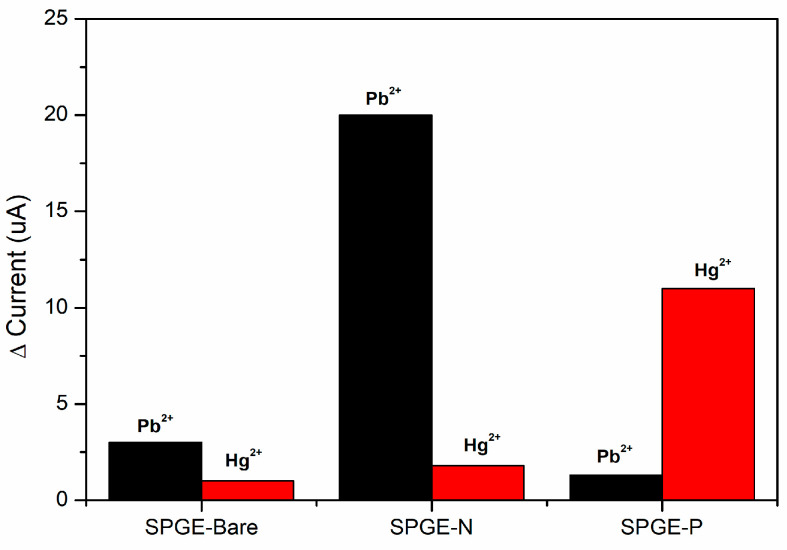
Electrochemical behavior of SPGE, **SPGE-N**, and **SPGE-P** in response to the presence of 10 nM of Pb^2+^ and Hg^2+^.

The above results, summarized in Figure 6, show the improved performance of the modified **SPGE-N** and **SPGE-P** electrodes, compared to SPGE, in selectively detecting Pb^2+^ and Hg^2+^ ions, respectively. In fact, for these ions, there is a marked improvement in the detection capability of over eight times for **SPGE-N** and ten times for **SPGE-P** when compared to the bare electrode.

The modified electrodes show the ability to detect heavy metal ions simultaneously and selectively when they are added at different concentrations, as illustrated in Figure 6. The peak current of the stripping reaction shows a proportional increase with the concentration, establishing a strong linear relationship in the concentration range between 1 nM and 10 nM for the metal ions under investigation. The ability to detect heavy metal ions at very low concentrations is essential for environmental applications.

**Figure 7:**
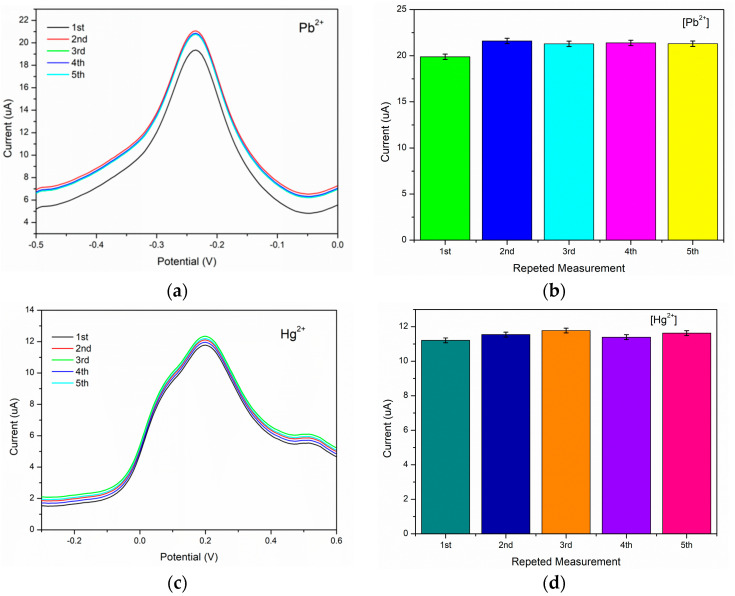
(**a**) Measurement curves of repeatability results obtained after several tests performed with the same **SPGE-N** sensor at 10 nM; (**b**) Histograms of repeatability results obtained after several tests with the same **SPGE-N** sensor at 10 nM; (**c**) Measurement curves of repeatability results obtained after several tests performed with the same SPGE-P sensor at 10 nM; (**d**) Histograms of repeatability results obtained after several tests with the same **SPGE-N** sensor at 10 nM.

The repeatability of the response of the modified electrodes to the addition of heavy metal ions was evaluated by performing five replicate tests in an acetate buffer solution (pH 4.5) with a final concentration of 10 nM for each metal. The results obtained and the average standard deviations for the oxidation peaks of SWASV are reported in Figure 7. The results showed that **SPGE-N** and **SPGE-P** have high and good repeatability for the detection of lead and mercury ions, respectively. The relative standard deviations (RSDs) evaluated after five experiments for Pb^2+^ and Hg^2+^ ions were 0.29 and 0.14, respectively. These RSD values of less than 1% for all tests performed indicate excellent repeatability. The LOD values were evaluated using the following formula:

(2) $$LOD=3\times \left(\frac{SD}{S}\right)$$

where *SD* is the standard deviation of three peak currents measured at the lowest concentration (50 ppb) and *S* is the slope of the calibration curve. The calculated limits of detection (LOD) were 0.41 nM and 0.035 nM for Pb^2+^ and Hg^2+^, respectively. The sensitivity of the sensors, computed as the slope of the calibration curve, were 5.84 µAnM^−1^cm^−2^ and 10 µAnM^−1^cm^−2^, respectively, for Pb^2+^ and Hg^2+^.

It is known that the analytical performance of pure gold thin-film electrodes with regard to heavy metal detection is not very high [64], but this ligand-selective chemical modification showed the potential to overcome this drawback.

The performances of the proposed sensors such as the linear range, sensitivity, and limit of detection were compared with the most recent reports in the literature (Table 1).

## 4. Real Samples Analyses

**Figure 8:**
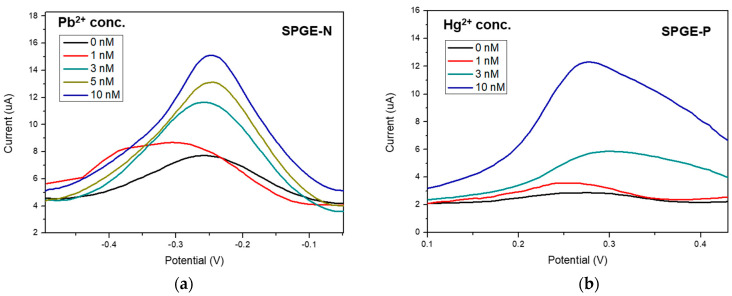
(**a**) SWASV of **SPGE-N** upon the addition of Pb^2+^ ions at ion concentrations of 0 and 10 nmol in real sample. (**b**) SWASV of **SPGE-P** to the addition of Hg^2+^ ions at ionic concentrations of 0 and 10 nmol in real sample.

The above-reported tests show that the developed modified platforms are very sensitive for the determination of toxic heavy metal ions at low traces. Therefore, we verified the efficiency of our electrodes in the detection of Pb^2+^ and Hg^2+^ in irrigation water by adding known amounts of the toxic heavy metal ions here investigated. The amounts used for contamination are below the maximum residue limits (MRLs) for these ions, as proposed by the EU directive. The procedure involved the detection of the two heavy metals analyzed in real water samples, previously diluted with acetate buffer (ratio 1:9, water/acetate buffer). The experiments were repeated three times, and the results obtained indicated that the recommended electrochemical method has the potential to accurately detect heavy metal ions in water samples. When compared to the response obtained in acetate buffer, the responses observed with real samples (Figure 8) confirmed that the sensor is not affected by the effect of the matrix.

Recovery values were evaluated as follows: Recovery (%) = (nM Pb^2+^ or Hg^2+^ found/NM Pb^2+^ or Hg^2+^ expected) × 100. The recovery values found, as shown in Table 2, were in the range of 96–132.2% for the lead ions on the **SPGE-N** electrode and were between 92.6 and 104.3% for mercury ions on the **SPGE-P** electrode. The experiments were repeated three times, and high recoveries were obtained for all the heavy metal ions investigated in this study. 

Furthermore, the stability of the sensors was evaluated by performing tests after storing the sensors for 6 months under ambient conditions. The **SPGE-N** and **SPGE-P** electrodes retained 92.6% and 93.4% of the initial response, thus indicating good long-term stability.

## 5. Conclusions

In this work, commercially available screen-printed gold electrodes have been functionalized via thiol chemistry with organic ligands able to selectively bind Hg^2+^ or Pb^2+^ ions for the electrochemical determination of these toxic ions in water. Square-wave anodic stripping voltammetry was applied for the electrochemical detection of these ions, either alone or in their mixtures. SWASV analyses revealed a higher detection capability of the functionalized electrodes when compared to bare SPGE. Better selectivity for Pb^2+^ ions was recorded for **SPGE-N**, while **SPGE-P** demonstrated detection capability for Hg^2+^ ions at very low concentrations (LOD of 0.41 nM for Pb^2+^ and 35 pM for Hg^2+^). The results of this study demonstrated, for the first time, the improved detection ability of SPGEs towards heavy metals after the chemical modification of the work electrode with properly designed sensitive elements. The reported results represent a valuable starting point for the development of electrochemical sensors which are capable of monitoring toxic trace heavy metals in water using simply modified SPEs and anodic stripping voltammetry.

## Figures and Tables

**Table 1:** Performance of the modified **SPGE-N** and **SPGE-P** gold electrodes proposed in this study for the detection of Pb^2+^ and Hg^2+^, respectively. For comparison, the performances of previous screen-printed gold electrodes for the detection of heavy metal ions are also shown.

Electrodes	LOD (nM)	Linear Range (nM)	Analyte	Technique	Ref.
Screen-printed gold electrode	0.6	10–10,000	Hg^2+^	CV	[65]
Au-(Cys)PW	4.0	0.01–0.2	Pb^2+^	SWV	[66]
Screen-printed gold electrode	500	0–50,000	Pb^2+^	SWASV	[67]
PTO/Au	0.06	0.2–1	Hg^2+^	DPV	[68]
MCGE	7.1	1.7–9.3	Pb^2+^	DPSV	[69]
Au–TiO_2_ NPs/Chit/gold-modified electrode	1.0	5.0–400.0	Hg^2+^	DPSV	[70]
**SPGE-N**	0.41	0–10	Pb^2+^	SWASV	This work
**SPGE-P**	0.035	0–10	Hg^2+^	SWASV	This work

**Table 2:** Determination of Pb^2+^ or Hg^2+^ in a real sample.

Modified Electrodes	Spiked (nM)	Found (nM)	Recovery (%)	RSD (%)
**SPGE-N**	1 of Pb^2+^	1.16 of Pb^2+^	116.0	1.39 (*n* = 3)
**SPGE-N**	3 of Pb^2+^	2.88 of Pb^2+^	96.0	1.85 (*n* = 3)
**SPGE-N**	5 of Pb^2+^	6.61 of Pb^2+^	132.2	3.31 (*n* = 3)
**SPGE-N**	10 of Pb^2+^	10.47 of Pb^2+^	104.7	1.28 (*n* = 3)
**SPGE-P**	1 of Hg^2+^	0.93 of Hg^2+^	93.0	1.59 (*n* = 3)
**SPGE-P**	3 of Hg^2+^	2.78 of Hg^2+^	92.6	2.11 (*n* = 3)
**SPGE-P**	10 of Hg^2+^	10.43 of Hg^2+^	104.3	1.98 (*n* = 3)

## Data Availability

Data are contained within the article.

## Supplementary Materials

The following supporting information can be downloaded at https://www.mdpi.com/article/10.3390/s24154935/s1, NMR and elemental analysis data.
