# Supplementary material for: Modified Gold Screen-Printed Electrodes for the Determination of Heavy Metals
Source: Sensors (Basel). 2024 Jul 30;24(15):4935. doi: 10.3390/s24154935 (PMC11314839; doi:10.3390/s24154935)
Supplement: Supplementary file 1 [file sensors-24-04935-s001.zip › sensors-3103354-supplementary.pdf]

Supplementary Materials

to:

Modified gold-screen printed electrodes for the determination of heavy metals

Consuelo Celesti [1*], Salvatore Vincenzo Giofrè[2*], Claudia Espro[1], Laura Legnani[3], Giovanni Neri[1] and Daniela Iannazzo[1]

[1] Department of Engineering, University of Messina, Contrada Di Dio, I-98166 Messina, Italy; ccelesti@unime.it, espro@unime.it, gneri@unime.it, diannazzo@unime.it (C.C.), (C.E.), (G.N.) (D.I.)
[2] Department of Chemical, Biological, Pharmaceutical and Environmental Sciences, University of Messina, Viale Ferdinando Stagno D'Alcontres 31, I98166, Messina, Italy; sgiofre@unime.it (S.V.G)
[3] Department of Biotechnology and Biosciences, University of Milano Bicocca, Piazza della Scienza 2, 20126, Milano, Italy; laura.legnani@unimib.it (L.L.)
* Correspondence: ccelesti@unime.it; sgiofre@unime.it

NMR and elemental analyses data of compounds 3,5–8, *Tr-N, and TrP*

Prop-2-yn-1-yl 3-((4-(((tert-butoxycarbonyl)amino)methyl)phenyl)amino) propanoate ***(3)***.
^{1}H NMR (500 MHz, $CDCl_3$) δ = 7.09 (d, J = 8.0 Hz, 2H), 6.56 (d, J = 8.0 Hz, 2H), 5.28 (s, 1H), 4.76 (bs, 1H), 4.69 (d, J = 2.5 Hz, 2H), 4.17 (d, J = 5.7 Hz, 2H), 3.45 (t, J = 6.3 Hz,2H), 2.65 (t, J = 6.3 Hz, 2H), 2.48 (td, J = 2.5, 0.7 Hz, 1H), 1.44 (s, 9H). ^{13}C NMR (126 MHz,$CDCl_3$) δ = 171.33, 155.99, 146.04, 129.23, 127.83, 113.17, 79.42, 77.62, 75.23, 52.24, 47.08,32.35, 28.59. Anal. Calcd for $C_{18}H_{24}N_2O_4$: C, 65.04; H, 7.28; N, 8.43; found C, 65.01; H, 7.29; N, 8.41.

(9H-fluoren-9-yl)methyl (2-azidoethyl)carbamate ***(5)***.
^{1}H NMR (500 MHz, $CDCl_3$) δ = 7.82 – 7.73 (m, 2H), 7.66 – 7.53 (m, 2H), 7.44 – 7.38 (m, 2H), 7.35 – 7.30 (m, 2H), 5.09 (bs, 1H), 4.43 (d, J = 6.9 Hz, 2H), 4.22 (t, J = 6.9 Hz, 1H), 3.43 (t, J = 5.5 Hz, 2H), 3.39 – 3.32 (m, 2H). ^{13}C NMR (126 MHz, $CDCl_3$) δ = 156.06, 143.83, 141.10, 127.03, 126.68, 125.14, 120.02, 71.57, 50.32, 47.51, 40.11. Anal. Calcd for $C_{17}H_{16}N_4O_2$: C, 66.22; H, 5.23; N, 18.17; found C, 66.20; H, 5.24; N, 18.15.

(1-(2-((((9H-fluoren-9-yl)methoxy)carbonyl)amino)ethyl)-1H-1,2,3-triazol-4-yl)methyl 3-((4-(((tert-butoxycarbonyl)amino)methyl)phenyl)amino)propanoate ***(6)***.
^{1}H NMR (500 MHz, $CDCl_3$) δ = 7.76 (d, J = 7.5 Hz, 2H), 7.56 (d, J = 7.4 Hz, 2H), 7.44 (s, 1H), 7.40 (tdd, J = 7.5, 1.2, 0.6 Hz, 2H), 7.31 (t, J = 7.3 Hz, 2H), 7.06 (d, J = 8.1 Hz, 2H), 6.52 (d, J = 8.1 Hz, 2H), 5.23 (s, 2H), 5.11 (bs, 1H), 4.75 (bs, 1H), 4.47 – 4.38 (m, 4H), 4.22 – 4.11 (m, 3H), 3.65 (d, J = 5.8 Hz, 2H), 3.41 (t, J = 6.3 Hz, 2H), 2.60 (t, J = 6.3 Hz, 2H), 1.45 (s, 9H). ^{13}C NMR (126 MHz, $CDCl_3$) δ = 172.16, 156.48, 155.99, 146.90, 143.80, 142.76, 141.40, 128.90, 127.85, 127.15, 125.06, 120.10, 113.12, 79.33, 66.76, 57.70, 49.79, 47.27, 40.85, 39.58, 33.94, 28.51. Anal. Calcd for $C_{35}H_{40}N_6O_6$: C, 65.61; H, 6.29; N, 13.12; found C, 65.59; H, 6.28; N, 13.11.

(1-(2-((((9H-fluoren-9-yl)methoxy)carbonyl)amino)ethyl)-1H-1,2,3-triazol-4-yl)methyl 3-((4-(aminomethyl)phenyl)amino)propanoate ***(Tr-N)***.
^{1}H NMR (500 MHz, $CDCl_3$) δ = 7.81 (dd, J = 7.9, 1.2 Hz, 2H), 7.63 (dd, J = 7.7, 1.4 Hz, 2H), 7.46 (s, 1H), 7.42 – 7.36 (m, 2H), 7.31 – 7.28 (m, 2H), 6.91 – 6.85 (m, 2H), 6.55 – 6.50 (m, 2H), 5.21 (s, 2H), 4.41 – 4.33 (m, 4H), 4.19 – 4.09 (m, 3H), 3.64 – 3.60 (m, 2H), 3.40 – 3.37 (m, 2H), 2.60 – 2.57 (m, 2H). ^{13}C NMR (125 MHz, $CDCl_3$) δ =167.31, 156.57, 151.50, 144.89, 143.83, 141.19, 134.59, 128.55, 127.08, 126.68, 125.14, 121.51,120.02, 113.41, 71.15, 50.19, 47.49, 46.09, 45.95, 39.75. Anal. Calcd for $C_{30}H_{32}N_6O_4$: C, 66.65;H, 5.97; N, 15.55; found C, 66.64; H, 5.95; N, 15.56.

(1-(2-aminoethyl)-1H-1,2,3-triazol-4-yl)methyl 3-((4-(((tert-butoxycarbonyl)amino) methyl)phenyl)amino)propanoate ***(7)***.

^{1}H NMR (500 MHz, $CDCl_3$) δ = 7.62 (s, 1H), 7.08 (d, J = 8.2 Hz, 2H), 6.54 (d, J = 8.2 Hz, 2H), 5.24 (s, 2H), 4.42 – 4.27 (m, 2H), 4.21– 4.11 (m, 2H), 3.48 – 3.40 (m, 2H), 3.27 – 3.11 (m, 2H), 2.63 (t, J = 6.3 Hz, 2H), 1.44 (s, 9H).^{13}C NMR (126 MHz, $CDCl_3$) δ = 172.29, 156.02, 146.98, 142.79, 129.03, 128.11, 124.37, 113.20,79.33, 57.91, 53.42, 44.42, 41.90, 39.65, 34.04, 28.56. Anal. Calcd for $C_{20}H_{30}N_6O_4$: C, 57.40; H,7.23; N, 20.08; found C, 57.38; H, 7.22; N, 20.05.

(1-(2-(((diethoxyphosphoryl)methyl)amino)ethyl)-1H-1,2,3-triazol-4-yl)methyl 3-((4-(((tert-butoxycarbonyl)amino)methyl)phenyl)amino)propanoate ***(8)***.

^{1}H NMR (500 MHz, $CDCl_3$) δ= 7.68 (s, 1H), 7.06 (d, J = 8.3 Hz, 2H), 6.54 (d, J = 8.3 Hz, 2H), 5.22 (s, 2H), 4.84 (bs, 1H), 4.39(t, J = 5.8 Hz, 2H), 4.18 – 4.02 (m, 4H), 3.88 (d, J = 6.3 Hz, 2H), 3.42 (t, J = 6.3 Hz, 2H), 3.16 (t,J = 5.9 Hz, 2H), 2.95 (d, J = 12.1 Hz, 2H), 2.61 (t, J = 6.3 Hz, 2H), 2.06 (bs, 1H), 1.43 (s, 9H), 1.36 – 1.25 (m, 6H). ^{13}C NMR (126 MHz, $CDCl_3$) δ = 172.23, 156.01, 146.98, 142.65, 128.99, 124.52, 113.17, 79.43, 62.63 (d, J = 6.7 Hz), 62.37 (d, J = 6.7 Hz), 57.84, 56.54, 44.73 (d, J =155.5 Hz), 39.59, 33.97, 29.78, 16.60. Anal. Calcd for $C_{25}H_{41}N_6O_7P$: C, 52.81; H, 7.27; N, 14.78;found C, 52.80; H, 7.26; N, 14.77.

(1-(2-(((diethoxyphosphoryl)methyl)amino)ethyl)-1H-1,2,3-triazol-4-yl)methyl 3-((4-(aminomethyl)phenyl)amino)propanoate ***(Tr-P)***.

^{1}H NMR (500 MHz, $CDCl_3$) δ = 7.68 (s, 1H), 7.09 (d, J= 8.5 Hz, 2H), 6.55 (d, J = 8.5 Hz, 2H), 5.24 (d, J = 0.5 Hz, 2H), 4.46 – 4.33 (m, 2H), 4.21 – 4.04(m, 4H), 3.72 (d, J = 8.0 Hz, 2H), 3.45 (t, J = 6.2 Hz, 2H), 3.25 – 3.11 (m, 2H), 3.02 – 2.86 (m,2H), 2.70 – 2.58 (m, 2H), 1.81 (bs, 2H), 1.40 – 1.29 (m, 6H). ^{13}C NMR (126 MHz, $CDCl_3$) δ =171.82, 147.28, 143.26, 141,16, 129.62, 124,65, 113.23, 62.69, 57.98, 5104, 50.14, 47.07, 45.18(d, J = 95.2 Hz), 39.59, 32.06, 16.36. Anal. Calcd for $C_{20}H_{33}N_6O_5P$: C, 51.27; H, 7.10; N, 17.94;found C, 51.26; H, 7.09; N, 17.94.
